# A Non-Invasive Method for Detection of Antihypertensive Drugs in Biological Fluids: The Salivary Therapeutic Drug Monitoring

**DOI:** 10.3389/fphar.2021.755184

**Published:** 2022-01-05

**Authors:** Valeria Avataneo, Elvira Fanelli, Amedeo De Nicolò, Franco Rabbia, Alice Palermiti, Marco Pappaccogli, Jessica Cusato, Francesco Giuseppe De Rosa, Antonio D'Avolio, Franco Veglio

**Affiliations:** ^1^ Laboratory of Clinical Pharmacology and Pharmacogenetics, Department of Medical Sciences, Amedeo di Savoia Hospital, University of Turin, Turin, Italy; ^2^ Division of Internal Medicine and Hypertension Unit, Department of Medical Sciences, A.O.U. Città Della Salute e Della Scienza di Torino, University of Turin, Turin, Italy; ^3^ Division of Infectious Diseases, Department of Medical Sciences, A.O.U. Città Della Salute e Della Scienza di Torino, University of Turin, Turin, Italy

**Keywords:** antihypertensive drugs, hypertension, liquid chromatography, tandem mass spectrometry, therapeutic drug monitoring

## Abstract

**Objectives:** Arterial hypertension is still the most frequent cause of cardiovascular and cerebrovascular morbidity and mortality. Antihypertensive treatment has proved effective in reduction of cardiovascular risk. Nevertheless, lifestyle interventions and pharmacological therapy in some cases are ineffective in reaching blood pressure target values, despite full dose and poly-pharmacological treatment. Poor adherence to medications is an important cause of treatment failure. Different methods to assess therapeutic adherence are currently available: Therapeutic drug monitoring in biological fluids has previously demonstrated its efficacy and reliability. Plasma and urine have been already used for this purpose, but they may be affected by some practical limitations. Saliva may represent a feasible alternative.

**Methods:** Fourteen antihypertensive drugs and two metabolites were simultaneously tested in plasma, urine, and saliva. Tested molecules included: atenolol, nebivolol, clonidine, ramipril, olmesartan, telmisartan, valsartan, amlodipine, nifedipine, doxazosin, chlorthalidone, hydrochlorothiazide, indapamide, sacubitril, ramiprilat, and sacubitrilat. Therapeutic drug monitoring was performed using ultra-high performance liquid chromatography, coupled to tandem mass spectrometry (UHPLC-MS/MS). The method has been preliminarily evaluated in a cohort of hypertensive patients.

**Results:** The method has been validated according to US Food and Drug Administration (FDA) and European Medicines Agency (EMA) guidelines. The application on a cohort of 32 hypertensive patients has demonstrated sensibility and specificity of 98% and 98.1%, respectively, with a good feasibility in real-life clinical practice.

**Conclusion:** Saliva may represent a feasible biological sample for therapeutic drug monitoring by non-invasive collection, prompt availability, and potential accessibility also in out-of-clinic settings.

## Introduction

Arterial hypertension is the leading cause of cardiovascular and cerebrovascular morbidity and mortality, involving about 30% of the worldwide population (Mills et al., 2020). Arterial hypertension treatment, including lifestyle interventions and pharmacological therapy, has the potential to prevent hypertension-mediated organ damage and reduce associated morbidity and mortality (Forouzanfar et al., 2017).

Nonetheless, blood pressure (BP) values are beyond target levels in 50% of treated subjects in high-income countries and 75% in low and middle-income countries, leading to uncontrolled, refractory or resistant hypertension (Burnier and Egan, 2019; Veglio and Mulatero, 2021). Poor adherence to antihypertensive medications is an important cause of treatment failure, involving about 50% of hypertensive patients (Vrijens et al., 2008; Avataneo et al., 2018). It can affect initiation of antihypertensive therapy, constancy of drugs assumption, and long-term persistence on treatment (Burnier and Egan, 2019). Suboptimal adherence has been associated to several adverse health outcomes, including myocardial infarction, chronic heart failure, stroke, end-stage renal disease, and overall mortality (Simpson et al., 2006; Perreault et al., 2009; Chowdhury et al., 2013; Herttua et al., 2013; Roy et al., 2013). Furthermore, it has important socio-economic implications, as it decreases the cost-effectiveness of health interventions, leading to poor clinical outcomes with increased costs for public health (Cherry et al., 2009).

The evaluation of therapeutic adherence is a clinical challenge, and different methods are currently available, including both direct and indirect procedures (Rabbia et al., 2016). Indirect methods are less invasive but less specific and with variable costs, including patient interview, diaries, questionnaires, pill count, review of prescriptions, and electronic monitoring. Conversely, direct methods are more invasive and expensive but more specific; they include direct observation of the patient during therapy administration and the measurement of drug and/or its metabolites in biological fluids. The latter method is generally defined as therapeutic drug monitoring (TDM), and it has proved to be the most accurate and cost-effective method for evaluating therapeutic adherence (Mackenzie and MacDonald, 2019). Furthermore, the use of multidrug TDM analysis, with measurement of several antihypertensive drugs simultaneously, allows to distinguish partial and complete non-adherence in clinical practice. The standard TDM biological matrices are currently liquid plasma and, to a lesser extent, whole blood. Nonetheless, plasma collection has some practical limitations: it can be invasive for the patient and not promptly available in out-of-clinic settings, requiring specialized personnel. Furthermore, shipment and processing are often expensive, discouraging TDM diffusion; additionally, plasma or blood concentration could not actually reflect effective drug concentration in the pharmacologically active site (Avataneo et al., 2019). At the same time, the therapeutic ranges of plasma concentrations for anti-hypertensive drugs are poorly described, preventing the use of TDM for dose adjustment. Nevertheless, the TDM of antihypertensive drugs retains a semi-quantitative value for the estimation and verification of therapeutic adherence.

For these reasons, several alternative matrices have been studied, in order to obtain less invasive and expensive techniques. The most frequently used alternative biological sample is urine, for its easy collection and cheaper processing. Many drugs and metabolites are excreted in the urinary system, allowing their detection in urine. Nevertheless, urinary TDM is currently considered mainly a qualitative technique because of high variability of pharmacokinetics between and within subjects, depending on renal function, urinary pH, and diuretic output (Avataneo et al., 2019). Dried blood spots TDM was also described, with collection of a drop of blood after finger pricking with a lancet (Peeters et al., 2020). Nevertheless, the accuracy of this technique can be largely affected by interindividual variability of hematocrit and by some drug characteristics, such as protein binding rate and penetration ability in red blood cells (O’Mara et al., 2011).

More specifically for TDM of anti-hypertensive drugs, the most frequently used matrices in previous studies are plasma and urine (Jung et al., 2013; Štrauch et al., 2013; Helfer et al., 2015; De Nicolò et al., 2016, 2017a; Pankaj et al., 2017).

Saliva is a biological matrix of easy collection and storage; its sampling is non-invasive for the patient, and it can be collected also by non-specialized staff, in out-of-clinic setting. These features allow multiple sampling over time, and its prompt collection has the potential to limit white coat adherence. Furthermore, it has been proposed as an alternative matrix to urine for toxicologic tests, for lower probability of voluntary adulteration of the patient (Wille et al., 2014). Saliva samples can be obtained by direct collection, by passive drool, or by use of specific devices.

In this study we aimed 1) to develop a method for detecting antihypertensive drugs in salivary samples, by using an ultra-high performance liquid chromatography technique, coupled to tandem mass spectrometry (UHPLC-MS/MS); 2) to validate this method, according to US Food and Drug Administration (FDA) and European Medicines Agency (EMA) guidelines (EMA, 2011; FDA, 2013, 2018); and 3) to preliminarily evaluate its feasibility as TDM technique in a small cohort of hypertensive patients.

## Materials and Methods

### Sample Collection and Preparation

Salivette® (Sarstedt, Nümbrecht, Germany) devices were used for saliva collection. The device is composed of a double external tube and an internal swab. For saliva collection, subjects introduced the swab in the mouth for 60 s and subsequently replaced it in the double tube system. Saliva collection was unstimulated and conducted in fasting condition for at least 30 min. The Salivette® was centrifugated for 2 min at 1,000 × *g*, in order to obtain a clear saliva sample. Each sample was stored in the dark at −20°C temperature until analysis.

An analytical method has been developed in order to detect the following drugs and metabolites: atenolol and nebivolol (ATE; NBV; β-blockers); clonidine (CLN; α2-agonist); doxazosin (DOX; α1-antagonist); amlodipine (AML; calcium antagonist); nifedipine (NFD; calcium antagonist); chlortalidone and hydrochlorothiazide (CHL; HCTZ; thiazide diuretics); indapamide (IDP; thiazide-like diuretic); ramipril (RAM; ACE-inhibitor); olmesartan, telmisartan, and valsartan (OLM; TEL; VAL; angiotensin-receptor blockers); and sacubitril (SCB; neprilysin inhibitor); furthermore, two drug metabolites were tested: ramiprilat (RAM-M) and sacubitrilat (SCB-M).

During the extraction procedure, for the calibration curve and the internal quality controls (QCs), 40 µl of internal standard (IS) working solution [prepared as follows: 6,7-dimethyl-2,3-di (2-pyridyl)quinoxaline (QX) at 500 ng/ml; (^2^H_7_)-atenolol at 250 ng/ml; and (^13^C_8_)-nifedipine, (^2^H_4_)-amlodipine, and (^13^C,^2^H_3_)-telmisartan at 25 ng/ml] were added to 40 µl of “calibrating” solution and 200 µl of blank saliva (for further details, see Stock solutions, Internal Standard, Standards, and Quality Controls—Online Supplementary). On the other hand, patients' samples were extracted by adding 40 µl of IS working solution to 40 µl of a blank mixture of water:acetonitrile (H_2_O:ACN) 90:10 (v:v) and 200 µl of saliva sample. Then, for the protein precipitation step, 1 ml of pure ACN was added to each sample, and, in order to equilibrate the salivary pH, 200 µl of ammonium acetate buffer 10 mM (+0.1% acetic acid) was added to each Eppendorf tube. Finally, all the samples were vortex-mixed and centrifuged at 21,000 × *g* at +4°C temperature for 10 min, then transferred into glass tubes and evaporated to dryness at 50°C (in about 1.5 h). Dry extracts were then re-suspended in 200 µl of H_2_O:ACN (+formic acid 0.05%) 90:10 (v:v), vortex-mixed, and finally transferred into total recovery vials; 7 µl of the resulting extracts are injected into the UHPLC-MS/MS system.

Before these procedures, in the preliminary phase of the project, experiments have been performed in order to evaluate drug retention by Salivette® matrix, using both saliva samples and solvent (see Preliminary experiments—Online Supplementary).

### Ultra-High Performance Liquid Chromatography-Mass Spectrometry/Mass Spectrometry Instruments and Chromatographic Conditions

A Perkin Elmer LX-50® UHPLC system coupled with Triple Quadrupole QSight 220® (Perkin Elmer, Milan, Italy) was used for the analysis. Chromatographic separation was performed through an Acquity® Ultra Performance Liquid Chromatography—High Strength Silica (UPLC HSS) T3 1.8 µm, 2.1 × 150 mm (Waters, Milan, Italy), protected by a frit (0.2 µm, 2.1 mm) (Waters, Milan, Italy) precolumn, at 40°C using a column thermostat, with a gradient of two mobile phases: phase A (H_2_O + formic acid 0.05%) and phase B (ACN + formic acid 0.05%); for further details, see Supplementary Table S1s—Online Supplementary. The instrument was settled in positive electrospray ionization mode (ESI+) for all drugs, except for HCTZ and CHL, which were detected in negative ionization mode (ESI−). General mass settings and multiple reaction monitoring (MRM) traces are shown in Supplementary Table S2s– Online Supplementary.

### Plasma and Urine Samples’ Collection and Analysis

Plasma and urine were stored at −20°C until analysis; these samples were analyzed following previously published methods, validated according to FDA guidelines (De Nicolò et al., 2016; 2017a).

### Method Validation

The salivary method has been validated according to FDA and EMA guidelines (EMA, 2011; FDA, 2013, 2018; Lynch, 2016). Accuracy, imprecision, and limits of quantification have been defined according to six inter-day validation sessions. Intra-day imprecision was evaluated in five intra-day replicates. Imprecision was expressed as the relative standard deviation (RSD) at each QC concentration (H, high; M, medium; L, low). Integration was performed by considering peak areas for each analyte. Specificity and selectivity were evaluated using six individual sources of the blank saliva matrix, individually analyzed, and evaluated for interferences. The upper limit of quantification (ULOQ) corresponds to standard 9 (STD9), the highest point of the calibration curve, for all the analytes; lower limits of quantification (LLOQ) were the lowest concentration of analytes in a sample which can be reliably quantified, with a deviation from the nominal concentration (measure of accuracy) and RSD (measure of precision) lower than 20% and with a signal-to-noise ratio higher than 5. The intermediate standards for the calibration curve were produced through serial 1:1 dilutions of the STD9 up to the LLOQ/STD1 (Table 1).

**TABLE 1:** Summary of drug concentrations in standards and quality control samples for each drug.

	STD 9	QC H	QC M	QC L	STD 1 (LLOQ)	LOD
CLN (ng/ml)	10	8	1	0.1	0.04	0.02
DOX (ng/ml)	10	8	1	0.1	0.04	0.02
NBV (ng/ml)	10	8	1	0.1	0.04	0.02
AML (ng/ml)	20	16	2	0.2	0.08	0.04
HCTZ (ng/ml)	100	80	10	1	0.39	0.19
NFD (ng/ml)	100	80	10	1	0.39	0.19
IDP (ng/ml)	100	80	10	1	0.39	0.19
TEL (ng/ml)	100	80	10	1	0.39	0.19
RAM (ng/ml)	100	80	10	1	0.39	0.19
OLM (ng/ml)	250	200	25	2.5	0.98	0.49
ATE (ng/ml)	1,000	800	100	10	3.91	1.91
CHL (ng/ml)	1,000	800	100	10	3.91	1.91
SCB (ng/ml)	2,000	1,600	200	20	7.81	0.98
VAL (ng/ml)	3,000	2,400	300	30	11.72	0.73
RAM-M (ng/ml)	100	80	10	1	0.39	0.09
SCB-M (ng/ml)	8,000	6,400	800	80	31.25	1.95
STD 9, standard 9, the highest point of the calibration curve; QC H, quality control high; QC M quality control medium; QC L, quality control low; STD 1, standard 1, the lowest point of the calibration curve; LLOQ, lower limit of quantification; LOD, limit of detection; CLN, clonidine; DOX, doxazosin; NBV, nebivolol; AML, amlodipine; HCTZ, hydrochlorothiazide; NFD, nifedipine; IDP, indapamide; TEL, telmisartan; RAM, ramipril; OLM, olmesartan; ATE, atenolol; CHL, chlortalidone; SCB, sacubitril; VAL, valsartan; RAM-M, ramiprilat; SCB-M, sacubitrilat.						

The lower limit of detection (LOD) was determined through further dilution of the LLOQ to obtain a minimum signal-to-noise ratio of 3, representing the minimum concentration which can be clearly distinguished from blank samples.

Recovery (REC) was evaluated during six validation sessions at high, medium, and low concentrations by comparing peaks areas from extracted QCs (pre-spiked) with those obtained by the direct injection of a chemical mix containing both the drugs and the IS at the same concentrations as the QCs. The extraction efficiency (EE) was measured by comparing the peak areas of pre-spiked QCs and post-spiked samples (blank saliva extracted and spiked with the drugs only before the analysis, to avoid possible degradations, at the same concentrations as the QCs).

Separate saliva samples from six healthy untreated donors were used for the extraction procedure and for the evaluation of matrix effect (ME). The ME was calculated by comparing the signal from the analysis of post-spiked samples at high, medium, and low levels with the ones from the direct injection of a chemical mix at the same concentration, as described by Taylor et al. and in FDA guidelines (post-extraction addition method) (Taylor, 2005; FDA, 2013). The IS-normalized matrix effect (IS-nME) was calculated as previously described by De Nicolò et al. (2017b).

### Patients' Selection

Hypertensive patients among those referred to Hypertension Centre of Turin, A.O.U. Città della Salute e della Scienza di Torino, were enrolled after expression of written informed consent. The inclusion criteria were as follows: age ≥ 18 years, established diagnosis of arterial hypertension, and ongoing antihypertensive treatment by at least 2 weeks before sampling. The exclusion criterion was any psychophysical condition or inability to perform samples' collection. Patients were in fasting condition for at least 30 min. Contextually with saliva sampling, a subset of patients agreed to perform a simultaneous collection of plasma and urine samples. The following antihypertensive drugs were tested, according to prescriptions: ATE, NBV, DOX, AML, NFD, CHL, HCTZ, RAM, OLM, TEL, and VAL; furthermore, a drug metabolite was tested (RAM-M).

The study was approved by Local Ethics Committee (TDM-TO Study, Protocol CS/504 September 03, 2015).

### Statistical Analysis

SAS 9.4 Software (SAS Institute Inc., United States) for Windows 10 was used for statistical analysis. Sensibility, specificity, and accuracy were calculated for salivary TDM, using plasma and urine as standard techniques of comparison, of proved reliability and accuracy.

## Results

### Method Validation

**FIGURE 1:**
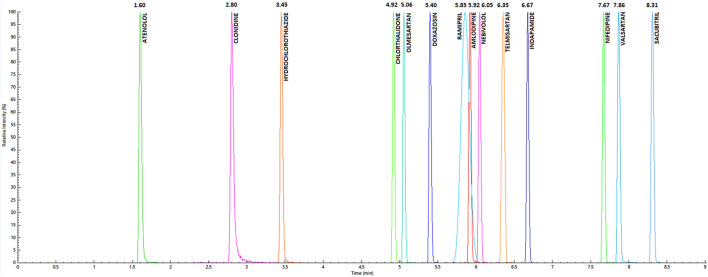
Chromatographic peaks and mean retention times for considered analytes, resulting from the injection of the highest point of the calibration curve.

Mean retention times (±0.05 min) of analytes are summarized in Figure 1. Calibration ranges and QC concentrations have been hypothesized according to those previously observed in plasma, then confirmed in the preliminary phase on real samples, and are summarized in Table 1. The LOD values resulted extremely low for most of the molecules, suitable for the detection of minimal concentrations in saliva. Analytes' accuracy and imprecision values at different concentrations are summarized in Table 2; all these parameters fit guidelines recommendations (EMA, 2011; FDA, 2013, 2018), showing mean bias and RSD lower than 15%. Exceptions were observed for the very low levels of AML, TEL, HCTZ, and NBV. REC, EE, ME, and IS-nME were in accordance with guidelines recommendations for almost all QC levels of all drugs and are shown in Table 2. In particular, the RSD values, which are considered the major sources of analytical inaccuracy and imprecision in UHPLC-MS/MS methods due to inter-sample variability in matrix composition and sample preparation procedures, were lower than 15% in most of cases.

**TABLE 2:** Summary of validation parameters.

		Accuracy (%)	Imprecision		Recovery mean (RSD)	Extraction efficiency mean (RSD)	Matrix effect mean (RSD)	IS-n-ME mean (RSD)
			Intra-day (RSD)	Inter-day (RSD)				
ATE	H	95.9	0.8	0.7	89.8 (4.3)	94.2 (5.1)	−4.5 (6.3)	−5.8 (3.4)
	M	95.4	1	1.0	90.0 (1.3)	90.5 (6.9)	−0.1 (7.7)	−3.2 (2.8)
	L	85.6	2.5	2.4	95.1 (4.7)	93.3 (8.9)	+2.6 (9.6)	−1.2 (5.0)
CLN	H	94.6	3.4	2.8	84.8 (1.3)	89.7 (4.7)	−5.2 (5.6)	−7.5 (6.5)
	M	91.9	3.5	3.2	85.8 (6.0)	89.0 (7.0)	−3.2 (7.9)	−5.2 (9.8)
	L	58.8	14	13.1	108.3 (18.2)	80.2 (10.2)	+36 (20.6)	+40.8 (13.2)
HCTZ	H	96.4	2.7	3.6	106.4 (2.4)	86.6 (10.9)	+23.8 (8.6)	+22.1 (9.2)
	M	108.2	0.9	1.3	135 (1.7)	92.9 (13.4)	+46.8 (11.4)	+48.5 (10.9)
	L	86.9	25.6	30.3	142.3 (2.2)	84.8 (8.2)	+69.1 (9.7)	+69.3 (9.6)
DOX	H	98.4	2.2	3.2	133.9 (1.2)	98.8 (0.4)	+35.6 (1.6)	+29.6 (0.6)
	M	109.5	4.3	6.8	149.6 (1.8)	103.3 (1.3)	+44.9 (3.2)	+41.9 (3.2)
	L	89.7	3.2	9.4	159.5 (14.0)	118.9 (11.0)	+33.9 (3.0)	+27.9 (3.8)
OLM	H	97.6	2.6	3.6	97.2 (2.1)	97.4 (4.3)	−0.1 (6.6)	−5.5 (16.0)
	M	93.5	2.3	3.4	108.9 (6.8)	97.0 (7.1)	+12.7 (12.7)	+8.0 (6.8)
	L	81.9	3.1	4.7	112.3 (8.8)	95.4 (9.3)	+18 (13.3)	+18.4 (10.0)
CHL	H	96.7	4.3	5.0	73.9 (7.4)	93.7 (12.8)	−20.2 (13.6)	−34.7 (6.8)
	M	97.1	3.8	5.0	79.6 (2.5)	95.0 (6.6)	−15.9 (11.8)	−19.5 (13.9)
	L	92.7	5.9	8.4	84.1 (15.5)	98.0 (8.6)	−13.4 (19.6)	−22.3 (16.2)
TEL	H	100.3	2.7	3.1	115.1 (8.4)	113.0 (7.1)	+2.2 (14.6)	−7.2 (6.3)
	M	97.9	3.8	8.0	118.7 (17.6)	120.0 (7.8)	−1.6 (12.7)	−4.9 (8.5)
	L	103.0	22.9	27.4	134.1 (34.3)	149.8 (31.2)	−9.6 (24.9)	−4.6 (22.1)
AML	H	93.7	3.7	7.4	96.3 (3.0)	88.9 (2.0)	+8.3 (1.2)	+4.7 (2.5)
	M	94.6	6.5	7.3	105.0 (6.3)	102.4 (20.0)	+13.8 (13.7)	+11.8 (2.4)
	L	73.2	35.1	32.9	94.0 (10.0)	101.1 (19.3)	−6.2 (9.4)	−16.8 (11.3)
RAM	H	101.9	2.3	2.2	85.1 (6.5)	92.8 (4.9)	−8.4 (5.8)	−13.6 (14.7)
	M	105.1	0.9	0.9	87.0 (6.1)	94.4 (3.0)	−7.9 (6.1)	−9.4 (10.1)
	L	90.0	2.7	4.2	89.5 (3.3)	99.6 (8.6)	−9.7 (8.6)	−6.3 (13.9)
RAM-M	H	101.6	2.3	3.5	94.9 (2.2)	95.2 (4.9)	−0.1 (5.9)	−4.7 (6.7)
	M	101.2	3.6	3.0	96.1 (7.2)	94.8 (8.6)	+1.7 (7.6)	−2.1 (9.3)
	L	78.1	4.7	5.1	108.5 (18.2)	96.8 (12.3)	+11.9 (12.6)	+7.6 (12.1)
NBV	H	89.1	3.2	10.6	102.3 (2.9)	99.8 (5.0)	+2.6 (2.2)	−1.4 (3.3)
	M	62.2	6.3	12.0	112.6 (4.2)	110.5 (8.4)	+2.5 (12.5)	−1.7 (12)
	L	51.7	4.2	10.0	108.6 (0.4)	95.9 (14.3)	+14.4 (14.8)	+13.5 (15.0)
NFD	H	109.9	7.2	8.7	102.6 (1.7)	124.7 (4.4)	−17.7 (2.7)	−20.5 (4.1)
	M	108.2	5.7	5.4	99.4 (4.3)	123.1 (4.7)	−19.1 (8.9)	−19.5 (20.1)
	L	100.3	10.8	10.4	118.5 (11.0)	125.0 (15.6)	−4.8 (4.7)	−11.2 (9.7)
SCB	H	100.4	2	2.2	93.8 (2.9)	100.4 (2.7)	−6.5 (1.2)	−9.3 (1.4)
	M	101.6	0.6	0.5	64.9 (1.9)	98.1 (2.5)	−3.3 (2.4)	−5.7 (3.6)
	L	113.6	1.2	1.4	96.7 (4.3)	97.5 (5.9)	−0.7 (4.6)	−2.8 (2.8)
SCB-M	H	93.4	1.2	1.8	92.5 (1.6)	97.0 (1.0)	−4.6 (1.2)	−7.8 (2.3)
	M	110.1	0.3	0.5	87.8 (2.1)	93.9 (2.0)	−6.5 (3.4)	−9.2 (3.9)
	L	107.2	1.8	1.9	89.3 (7.1)	95.2 (6.0)	−6.3 (8.3)	−9.9 (6.5)
IDP	H	97.8	2.1	1.7	79.5 (5.6)	88.3 (5.9)	−9.9 (6.5)	−14.7 (7.2)
	M	113.4	2.6	2.5	78.6 (7.4)	90.4 (6.6)	−13.0 (8.5)	−17.3 (9.0)
	L	110.4	5.6	5.7	84.9 (10.9)	99.6 (14.8)	−13.6 (13.7)	−16.1 (13.0)
VAL	H	99.0	3.2	2.7	91.7 (1.3)	96.5 (0.7)	−5.0 (0.9)	−14.9 (11.1)
	M	110.0	1.5	1.9	91.0 (2.9)	95.1 (1.2)	−4.2 (3.4)	−8.5 (10.9)
	L	89.6	1.2	2.0	92.4 (4.3)	94.7 (4.2)	−2.4 (3.9)	−1.4 (13.9)
RSD, relative standard deviation; IS-n-ME, internal standard-normalized matrix effect; ATE, atenolol; CLN, clonidine; HCTZ, hydrochlorothiazide; DOX, doxazosin; OLM, olmesartan; CHL, chlortalidone; TEL, telmisartan; AML, amlodipine; RAM, ramipril; RAM-M, ramiprilat; NBV, nebivolol; NFD, nifedipine; SCB, sacubitril; SCB-M, sacubitrilat; IDP, indapamide; VAL, valsartan; H, high; M, medium; L, low.								

Calibration curves fitted quadratic regression models by passing through the origin of the axes; a weighting factor 1/*X* was used to ensure high accuracy at low concentrations. Regression coefficients (r^2^) of calibration curves were all above 0.996.

### Recovery From the Salivette®

With the use of solvent, AML, TEL, DOX, and NBV showed a >90% concentration reduction after passage through Salivette® fiber; SCB, SCB-M, CLN, HCTZ, RAM, and ATE showed a reduction between 20% and 50%; finally, OLM, CHL, VAL, NFD, and RAM-M showed a <20% variation, consistent with a casual error. IDP showed a moderate increase, confirmed in both replicate experiments.

These observations were moderately confirmed also in saliva matrix: AML, TEL, DOX, NBV, and also HCTZ showed a >50% loss after the passage through Salivette® fiber; CHL, NFD, and ATE showed a reduction by 20% and 50%; finally, OLM, SCB, SCB-M, VAL, RAM, and RAM-M showed a <20% variation, consistent with a casual error. IDP and CLN showed an increase in concentrations. The abovementioned results are detailed in Table 3; some molecule-specific parameters (partition coefficient, logP; acid dissociation constant, pKa; molecular weight, MW; and chromatographic retention time, R-) were analyzed, but they did not present any correlations with drugs retention (Table 3).

**TABLE 3:** Summary of results about drug retention by Salivette^®^ fiber.

	Mean solvent loss (%)	Mean saliva loss (%)	logP	pKa	MW	RT
AML	−94	−97	3.00	8.60	408.9	5.92
TEL	−100	−62	7.70	3.86	514.6	6.35
DOX	−99	−100	2.10	6.52	451.5	5.40
NBV	−97	−98	4.18	8.13	405.4	6.05
HCTZ	−33.5	−54	−0.07	7.90	297.7	3.45
ATE	−46.5	−21	0.16	9.60	266.34	1.60
SCB	−30	−14	3.90	4.18	411.5	8.31
RAM	−31	2	2.90	3.74	416.5	5.85
OLM	−1.5	2	0.73	4.30	446.5	5.06
CHL	−14	−30	0.85	9.36	338.8	4.92
CLN	−28.5	24	1.59	8.05	230.1	2.80
IDP	19.5	33	2.52	8.80	365.8	6.67
VAL	−13	−10	1.50	4.73	435.5	7.86
NFD	−13	−38	2.20	3.93	346.3	7.67
RAM-M	−12	13	0.54	n.a	388.5	5.00
SCB-M	−22.5	−2	1.99	n.a	383.4	7.04
LogP, partition coefficient; pKa, acid dissociation constant; MW, molecular weight; RT, retention time; AML, amlodipine; TEL, telmisartan; DOX, doxazosin; NBV, nebivolol; HCTZ, hydrochlorothiazide; ATE, atenolol; SCB, sacubitril; RAM, ramipril; OLM, olmesartan; CHL, chlortalidone; CLN, clonidine; IDP, indapamide; VAL, valsartan; NFD, nifedipine; RAM-M, ramiprilat; SCB-M, sacubitrilat						

### Patients' Samples Analysis

Thirty-two patients were enrolled for method evaluation in real-life clinical practice. The mean age of subjects was 59 ± 18 years, with 69% males. Forty-six percent of patients were on therapy with more than one antihypertensive drug.

In a subset on 27 patients, whose adherence had been previously verified through plasma analysis, paired analyses have been considered, and the saliva–plasma ratio (S/P Ratio) has been measured, aiming to evaluate the overall method performance in terms of concordance for the determination of adherence profiles. This sub-analysis demonstrated that most determinations (48 out of a total of 53 matches) resulted fully confirmed, suggesting overall sensibility and specificity of 98% and 98.1%, respectively, and an overall accuracy of 98.1%.

Furthermore, a preliminary analysis on the simultaneous quantification of the 14 drugs across the three different matrices (saliva, plasma, and urine spot) was conducted on a subset of 24 patients. Of these, 12 had a prescription for AML; 9 for TEL; 5 for RAM; 4 for DOX; 3 for NBV; 2 for OLM, NFD, HCTZ, CHL, and VAL; 1 for ATE; and none for IDP, SCB, and CLN.

In 50 total matches, 39 were fully confirmed across the three different matrices, and 48 were confirmed on two of three.

Additionally, saliva demonstrated a higher sensibility for NFD and TEL detection when compared to urine.

Data on comparison among saliva, plasma, and urine are extensively reported in Table 4.

**TABLE 4:** Summary of saliva, plasma, and urine analysis.

	Saliva			Plasma			Urine			S/P ratio		S-P correlation
	n	Median (ng/ml)	IQR	n	Median (ng/ml)	IQR	n	Median (ng/ml)	IQR	Median	IQR	R (*p*-value)
ATE	1	38.60	n.a	1	100.72	n.a	1	4,839.48	n.a	0.38	n.a	n.a
NBV	6	0.74	0.18–8.77	3	0.57	0.29–21.17	3	0.63	n.d.—51.37	9.50	4.76–14.24	−0.448; 0.704
AML	21	6.13	2.92–9.24	14	6.29	4.15–15.02	12	177.93	63.48–633.87	0.67	0.40–1.49	0.308 (0.285)
NFD	2	1.77	1.51–2.02	2	36.22	34.42–38.03	2	n.d	n.a	0.05	0.04–0.06	n.a
DOX	6	3.01	1.26–4.44	4	7.12	2.44–12.45	4	55.11	23.68–99.49	0.32	0.26–0.40	0.745 (0.255)
OLM	7	0.22	0.12–0.51	3	244.94	129.38–363.32	2	1,039.36	679.36–1,443.64	<0.01	0.00–0.00	0.259 (0.833)
TEL	13	2.60	0.49–26.84	9	45.82	23.02–53.78	9	11.57	4.93–14.71	0.02	0.00–0.13	−0.047 (0.905)
VAL	2	2.19	1.27–3.12	2	749.78	562.92–936.63	2	2,094.50	1,204.25–2,984.75	<0.01	0.00–0.00	n.a
RAM	6	0.13	0.09–0.20	5	0.73	0.68–2.56	5	9.30	0.95–21.22	0.10	0.06–0.18	−0.146 (0.815)
RAM-M	6	0.11	0.10–0.27	5	11.12	4.16–13.83	5	384.26	324.40–636.82	0.01	0.01–0.01	0.126 (0.840)
HCTZ	6	6.23	1.38–58.20	2	10.82	7.80–13.83	2	538.22	3,970.63–6,552.00	10.48	5.28–15.68	n.a
CHL	4	5.35	3.17–17.27	3	50.50	35.69–110.30	3	906.77	491.40–1,322.13	0.17	0.16–0.23	0.993 (0.075)
ATE, atenolol; NBV, nebivolol; AML, amlodipine; NFD, nifedipine; DOX, doxazosin; OLM, olmesartan; TEL, telmisartan; VAL, valsartan; RAM, ramipril; RAM-M, ramiprilat; HCTZ, hydrochlorothiazide; CHL, chlortalidone; S/P ratio, saliva/plasma ratio; IQR, interquartile range; n.a., not available												

For some drugs (OLM, VAL, RAM, and RAM-M), the median measured saliva concentration was lower than the LLOQ but still higher than the limit of detection; in these cases, we considered it reliable (due to the good quality of the chromatographic peak and the absence of noise) but considering an error risk higher than 20%.

## Discussion

Therapeutic adherence in hypertensive patients is generally unsatisfactory, as previous studies have underlined (Avataneo et al., 2018; Burnier and Egan, 2019). It has relevant health and socio-economic implications, in terms of higher disability, morbidity, and overall mortality (Cherry et al., 2009). For these reasons, it is important to recognize lack of therapeutic adherence, especially among patients with difficult-to-treat or resistant hypertension. TDM of antihypertensive drugs has proven to be a valid and cost-effective instrument for this purpose (Burnier and Egan, 2019). The biological fluids most frequently used in previous studies were urine and blood, including plasma and dried blood spot (Jung et al., 2013; Štrauch et al., 2013; Helfer et al., 2015; De Nicolò et al., 2016, 2017a; Pankaj et al., 2017).

In the present study, the feasibility of a new antihypertensive TDM technique on salivary samples was evaluated, with preliminary application on a small cohort of hypertensive patients. The method was validated, according to FDA and EMA guidelines (EMA, 2011; FDA, 2018), resulting eligible for a quali-quantitative detection and quantification of extremely low drug concentrations in saliva. Furthermore, it demonstrated applicability in real-life clinical practice, with a good sensibility and specificity for determination of therapeutic adherence in the cohort of hypertensive patients in which it was applied.

A TDM technique of easy and prompt application has the potential to encourage a greater diffusion, including non-specialized clinics and out-of-clinic settings. Furthermore, a method of easy use allows limitation of “white coat adherence” that can occur with a programmed adherence evaluation: saliva collection can be easily performed and repeated without programming, improving detection of poor treatment adherence.

Another study previously analyzed the use of oral fluids for cardiovascular drugs therapeutic monitoring, but the described method was characterized by higher limits of quantification and detection (even 1,250×), that could potentially lead to a higher rate of false negatives, with overestimation of non-adherence rate (Richter et al., 2019). On the contrary, the present method has demonstrated a high analytical sensibility, with a LLOQ lower than 1 ng/ml for 11 of 16 tested molecules, other than extremely low LOD values.

The comparison of salivary concentrations and relative penetration from plasma analysis to plasma and urine has highlighted a good accuracy of this technique and an overall sensibility and specificity of 98% and 98.1%, respectively.

A very high inter-patient variability in saliva was observed, but this phenomenon could depend on water consumption, time from dosing, and salivary pH. In fact, several factors may influence drugs concentration in oral fluids, including molecular mass, lipophilicity, ionization state, and protein binding; additionally, retention from Salivette® fiber may reduce drug concentration in saliva. For this reason, the sensibility of the method is important in the perspective of TDM application, even when proposing a semi-quantitative technique, to avoid false-negative results (Burnier and Egan, 2019). Furthermore, saliva demonstrated a higher sensibility when compared to urine for some drugs, as NFD. It is probably due to photolability and higher degradation rate of this molecule in urine (Aman and Thoma, 2003). Similarly, it is likely that molecules with prevalent biliary excretion could be characterized by a higher detection power of salivary TDM, with a lower sensibility of urine. Nonetheless, the present study did not show significant difference in sensibility of urine and saliva for TEL, which has a prevalent biliary excretion.

A low S/P concentration ratio was observed for all OLM, TEL, and VAL, suggesting an influence of high protein binding rate and molecular mass of these sartans. This evidence further suggests that other matrices (mainly plasma or, alternatively, urine for OLM and VAL) should be preferred for TDM of sartans. Conversely, HCTZ and NBV showed a high relative penetration in saliva and, in the case of NBV, better performance for TDM purpose in saliva matrix when compared with urine. Also, AML showed a good penetration rate, probably influenced by its lipophilic and basic characteristics and low protein binding rate.

In this study, several antihypertensive drug classes were tested, but the progressive diffusion of single pill combinations may allow in the future the selection of only one drug of the combination to evaluate adherence, ideally preferring more stable and detectable molecules (for example AML, which is also one of the most used drugs in polypill formulations).

This study had some limitations: 1) the use of Salivette®, as of other absorbing materials, may result in partial absorption of drugs and contamination with other substances, interfering with the measuring procedure; however, in the present study this effect was not significant, in terms of reduction of sensibility of the technique; 2) the small cohort of subjects did not allow inference about pharmacokinetics variations dependent on age, sex, smoking, time of medications intake, or food interference; further studies are needed for characterization of these variables.

## Conclusion

A good adherence to prescribed medications is an essential prerequisite for a good BP control in hypertension. The development of new and easy-to-use methods for non-adherence screening is critical to identify the problem in clinical practice and improve adherence on a large scale.

The present study described a validated method for salivary TDM of anti-hypertensive drugs, also evaluating the comparative performance with other matrices that are classically used for the evaluation of treatment adherence of patients. Preliminary results in a small cohort of hypertensive patients showed better performance of saliva when compared to urine for NFD and NBV, while all tested sartans showed lower penetration rate in saliva.

Considering its extremely favorable features, the salivary TDM could become a common practice for fast, cheap, and large-scale quali-quantitative screening of patients' adherence to anti-hypertensive treatment.

## Data Availability

The raw data supporting the conclusion of this article will be made available by the authors on request, without undue reservation.

## Author’s Note

Laboratory of Clinical Pharmacology and Pharmacogenetics, Department of Medical Sciences, Amedeo di Savoia Hospital, University of Turin, Turin, Italy. PHASE I AIFA, UNI EN ISO 9001 and 13485 Certificate Laboratory, Certificate No. IT-64386 and DM/17/154/S. Certification for “Design, Development and Application of Determination Methods for Clinical Analytes and Drugs, also with Reference to *in vitro* Diagnostics, Pharmacogenetic Analyses” and “Design and Development and Application of Quantification and Detection Methods of Clinical Analytes and Drugs Finalized to the Production of *in vitro* Diagnostics” www.tdm-torino.org.

## References

[B1] Aman W. Thoma K. (2003). Particular Features of Photolabile Substances in Tablets. Pharmazie 58, 645–650. 14531462

[B2] Avataneo V. D'Avolio A. Cusato J. Cantù M. De Nicolò A. (2019). LC-MS Application for Therapeutic Drug Monitoring in Alternative Matrices. J. Pharm. Biomed. Anal. 166, 40–51. 10.1016/j.jpba.2018.12.040 30609393

[B3] Avataneo V. De Nicolò A. Rabbia F. Perlo E. Burrello J. Berra E. (2018). Therapeutic Drug Monitoring-Guided Definition of Adherence Profiles in Resistant Hypertension and Identification of Predictors of Poor Adherence. Br. J. Clin. Pharmacol. 84, 2535–2543. 10.1111/bcp.13706 29971815 PMC6177709

[B4] Burnier M. Egan B. M. (2019). Adherence in Hypertension. Circ. Res. 124, 1124–1140. 10.1161/CIRCRESAHA.118.313220 30920917

[B5] Cherry S. B. Benner J. S. Hussein M. A. Tang S. S. Nichol M. B. (2009). The Clinical and Economic Burden of Nonadherence with Antihypertensive and Lipid-Lowering Therapy in Hypertensive Patients. Value Health 12, 489–497. 10.1111/j.1524-4733.2008.00447.x 18783393

[B6] Chowdhury R. Khan H. Heydon E. Shroufi A. Fahimi S. Moore C. (2013). Adherence to Cardiovascular Therapy: a Meta-Analysis of Prevalence and Clinical Consequences. Eur. Heart J. 34, 2940–2948. 10.1093/eurheartj/eht295 23907142

[B7] De Nicolò A. Avataneo V. Rabbia F. Bonifacio G. Cusato J. Tomasello C. (2016). UHPLC-MS/MS Method with Protein Precipitation Extraction for the Simultaneous Quantification of Ten Antihypertensive Drugs in Human Plasma from Resistant Hypertensive Patients. J. Pharm. Biomed. Anal. 129, 535–541. 10.1016/j.jpba.2016.07.049 27497654

[B8] De Nicolò A. Avataneo V. Rabbia F. Sciandra M. Tosello F. Cusato J. (2017a). UHPLC-MS/MS Method with Sample Dilution to Test Therapeutic Adherence through Quantification of Ten Antihypertensive Drugs in Urine Samples. J. Pharm. Biomed. Anal. 142, 279–285. 10.1016/j.jpba.2017.05.018 28538203

[B9] De Nicolò A. Cantù M. D'Avolio A. (2017b). Matrix Effect Management in Liquid Chromatography Mass Spectrometry: The Internal Standard Normalized Matrix Effect. Bioanalysis 9, 1093–1105. 10.4155/bio-2017-0059 28737421

[B10] EMA (2011). Guideline on Bioanalytical Method Validation. Available at: https://www.ema.europa.eu/en/documents/scientific-guideline/guideline-bioanalytical-method-validation_en.pdf .

[B11] FDA (2013). Bioanalytical Method Validation Guidance for Industry.

[B12] FDA (2018). Bioanalytical Method Validation Guidance for Industry. Available at: https://www.fda.gov/files/drugs/published/Bioanalytical-Method-Validation-Guidance-for-Industry.pdf .

[B13] Forouzanfar M. H. Liu P. Roth G. A. Ng M. Biryukov S. Marczak L. (2017). Global Burden of Hypertension and Systolic Blood Pressure of at Least 110 to 115 Mm Hg, 1990-2015. JAMA 317, 165–182. 10.1001/jama.2016.19043 28097354

[B14] Gupta P. Patel P. Štrauch B. Lai Y. F. Gulsin G. S. Akbarov A. (2017). Biochemical Screening for Nonadherence Is Associated with Blood Pressure Reduction and Improvement in Adherence. Hypertension 70, 1042–1048. 10.1161/HYPERTENSIONAHA.117.09631 28847892 PMC5642335

[B15] Helfer A. G. Michely J. A. Weber A. A. Meyer M. R. Maurer H. H. (2015). Orbitrap Technology for Comprehensive Metabolite-Based Liquid Chromatographic-High Resolution-Tandem Mass Spectrometric Urine Drug Screening - Exemplified for Cardiovascular Drugs. Anal. Chim. Acta 891, 221–233. 10.1016/j.aca.2015.08.018 26388381

[B16] Herttua K. Tabák A. G. Martikainen P. Vahtera J. Kivimäki M. (2013). Adherence to Antihypertensive Therapy Prior to the First Presentation of Stroke in Hypertensive Adults: Population-Based Study. Eur. Heart J. 34, 2933–2939. 10.1093/eurheartj/eht219 23861328 PMC3791393

[B17] Jung O. Gechter J. L. Wunder C. Paulke A. Bartel C. Geiger H. (2013). Resistant Hypertension? Assessment of Adherence by Toxicological Urine Analysis. J. Hypertens. 31, 766–774. 10.1097/HJH.0b013e32835e2286 23337469

[B18] Lynch K. L. (2016). CLSI C62-A: A New Standard for Clinical Mass Spectrometry. Clin. Chem. 62, 24–29. 10.1373/clinchem.2015.238626 26430075

[B19] Mackenzie I. S. MacDonald T. M. (2019). Identifying Poor Adherence to Antihypertensive Medications in Patients with Resistant Hypertension. Br. J. Clin. Pharmacol. 85, 5–7. 10.1111/bcp.13806 30478934 PMC6303199

[B20] Mills K. T. Stefanescu A. He J. (2020). The Global Epidemiology of Hypertension. Nat. Rev. Nephrol. 16, 223–237. 10.1038/s41581-019-0244-2 32024986 PMC7998524

[B21] O'Mara M. Hudson-Curtis B. Olson K. Yueh Y. Dunn J. Spooner N. (2011). The Effect of Hematocrit and Punch Location on Assay Bias during Quantitative Bioanalysis of Dried Blood Spot Samples. Bioanalysis 3, 2335–2347. 10.4155/bio.11.220 22011181

[B22] Peeters L. E. J. Feyz L. Hameli E. Zwart T. Bahmany S. Daemen J. (2020). Clinical Validation of a Dried Blood Spot Assay for 8 Antihypertensive Drugs and 4 Active Metabolites. Ther. Drug Monit. 42. Available at:. 10.1097/FTD.0000000000000703 https://journals.lww.com/drug-monitoring/Fulltext/2020/06000/Clinical_Validation_of_a_Dried_Blood_Spot_Assay.17.aspx 31593031

[B23] Perreault S. Dragomir A. White M. Lalonde L. Blais L. Bérard A. (2009). Better Adherence to Antihypertensive Agents and Risk Reduction of Chronic Heart Failure. J. Intern. Med. 266, 207–218. 10.1111/j.1365-2796.2009.02084.x 19623691

[B24] Rabbia F. Fulcheri C. Di Monaco S. Covella M. Perlo E. Pappaccogli M. (2016). Adherence to Antihypertensive Therapy and Therapeutic Dosage of Antihypertensive Drugs. Cardiovasc. Prev. 23, 341–345. 10.1007/s40292-016-0158-z 27160721

[B25] Richter L. H. J. Jacobs C. M. Mahfoud F. Kindermann I. Böhm M. Meyer M. R. (2019). Development and Application of a LC-HRMS/MS Method for Analyzing Antihypertensive Drugs in Oral Fluid for Monitoring Drug Adherence. Anal. Chim. Acta 1070, 69–79. 10.1016/j.aca.2019.04.026 31103169

[B26] Roy L. White-Guay B. Dorais M. Dragomir A. Lessard M. Perreault S. (2013). Adherence to Antihypertensive Agents Improves Risk Reduction of End-Stage Renal Disease. Kidney Int. 84, 570–577. 10.1038/ki.2013.103 23698228

[B27] Simpson S. H. Eurich D. T. Majumdar S. R. Padwal R. S. Tsuyuki R. T. Varney J. (2006). A Meta-Analysis of the Association between Adherence to Drug Therapy and Mortality. Bmj 333, 15. 10.1136/bmj.38875.675486.55 16790458 PMC1488752

[B28] Štrauch B. Petrák O. Zelinka T. Rosa J. Šomlóová Z. Indra T. (2013). Precise Assessment of Noncompliance with the Antihypertensive Therapy in Patients with Resistant Hypertension Using Toxicological Serum Analysis. J. Hypertens. 31. Available at:. 10.1097/HJH.0b013e3283652c61 https://journals.lww.com/jhypertension/Fulltext/2013/12000/Precise_assessment_of_noncompliance_with_the.20.aspx 24220593

[B29] Taylor P. J. (2005). Matrix Effects: The Achilles Heel of Quantitative High-Performance Liquid Chromatography-Electrospray-Tandem Mass Spectrometry. Clin. Biochem. 38, 328–334. 10.1016/j.clinbiochem.2004.11.007 15766734

[B30] Veglio F. Mulatero P. (2021). Resistant or Refractory Hypertension: it Is Not Just the of Number of Drugs. J. Hypertens. 39, 589–591. 10.1097/HJH.0000000000002814 33534344

[B31] Vrijens B. Vincze G. Kristanto P. Urquhart J. Burnier M. (2008). Adherence to Prescribed Antihypertensive Drug Treatments: Longitudinal Study of Electronically Compiled Dosing Histories. BMJ 336, 1114–1117. LP. 10.1136/bmj.39553.670231.25 18480115 PMC2386633

[B32] Wille S. M. R. Baumgartner M. R. Fazio V. Di. Samyn N. Kraemer T. (2014). Trends in Drug Testing in Oral Fluid and Hair as Alternative Matrices. Bioanalysis 6, 2193–2209. 10.4155/bio.14.194 25383732

