# Supplementary material for: A Non-Invasive Method for Detection of Antihypertensive Drugs in Biological Fluids: The Salivary Therapeutic Drug Monitoring
Source: Front Pharmacol. 2022 Jan 5;12:755184. doi: 10.3389/fphar.2021.755184 (PMC8766966; doi:10.3389/fphar.2021.755184)
Supplement: Supplementary file 1 [file DataSheet1.docx]

Supplementary Material

# Supplementary data

## Chemicals

Ultra-High Performance Liquid Chromatography (UHPLC) grade acetonitrile (ACN) and methanol (MeOH) were purchased from VWR (Radnor, PA, USA). UHPLC grade H_2_O was produced with Milli-DI system coupled with a Synergy 185 system by Millipore (Milan, Italy). Blank saliva was obtained from healthy donors. Atenolol (ATE; purity >98%), clonidine hydrochloride (CLN; overall purity 86.22%), doxazosin mesylate (DOX; overall purity 81.29%), amlodipine (AML; purity 99.52%), nifedipine (NFD; purity 97.64%), chlorthalidone (CHL; purity 99.9%), hydrochlorothiazide (HCTZ; purity 99.2%), ramipril (RAM; purity 99.81%), telmisartan (TEL; purity 99.96%), Olmesartan (OLM; purity 99.01%), valsartan (VAL; purity 99.35%), nebivolol hydrochloride (NBV; overall purity 90.83%), sacubitril (SCB; purity 99.71%), sacubitrilat (SCB-M; purity 99.85%), indapamide (IDP; purity 99.61%), were purchased from MedChem Express (Monmouth Junction, NJ, USA); ramiprilat (RAM-M; purity 99.4%) was purchased from Santa Cruz Biotecnology (Dallas, TX, USA). The internal standards (IS), 6,7-dimethyl-2,3-di(2-pyridyl)quinoxaline (QX; purity 98.5%) was purchased from MedChem Express, [^2^H_4_]-amlodipine maleate (overall purity 78%), [^13^C_8_]-nifedipine (purity 95.6%), [^13^C,^2^H_3_]-telmisartan (purity 98.4%) were purchased from Alsachim (Illkirch, France) and [^2^H_7_]-atenolol (purity 98.7%) was purchased from Sigma-Aldrich (Saint Louis, MO, USA).

All powders were stored at -20°C or +4°C in the dark, according to the manufacturer’s recommendations, in order to prevent any possible degradation.

## Stock solutions, Internal Standard, Standards, and Quality Controls

Stock solutions (1 mg/mL) were prepared singularly as below: DOX, AML, [^2^H_4_]-amlodipine, CHL, HCTZ, NFD, [^13^C_8_]-nifedipine, OLM, RAM, RAM-M, SCB-M, IDP, VAL and QX in a mixture of H_2_O:MeOH 5:95 (v:v); TEL and [^13^C,^2^H_3_]-telmisartan in dimethyl sulfoxide (DMSO):MetOH 5:95 (v:v); ATE, [^2^H_7_]-atenolol and NBV in H_2_O:MetOH 50:50 (v:v), CLN in pure H_2_O and SCB in H_2_O:MetOH:DMSO 1:1:1 (v:v:v).

Stock solutions were stored at -20°C until use (less than 6 months). Single aliquots of “calibrating” Standard (STD) 9 and Quality Controls (QCs) were prepared by independently spiking a mixture of H_2_O:ACN 90:10 with stock solutions and then stored at -20°C. IS working solution was prepared by diluting 2 μL of QX, 1 μL of [^2^H_7_]-atenolol, 0.1 μL of [^13^C_8_]-nifedipine, [^2^H_4_]-amlodipine and [^13^C,^2^H_3_]-telmisartan stock solution in 4 mL of H_2_O:MetOH [50:50] at each analytical session (final concentrations are as follow: 500 ng/mL for QX, 250 ng/mL for [^2^H_7_]-atenolol and 25 ng/mL for [^13^C_8_]-nifedipine, [^2^H_4_]-amlodipine and [^13^C,^2^H_3_]-telmisartan).

## Stability

According to this protocol, standards and quality controls (QCs) were prepared in a solvent very similar to the ones used for stock solutions preparation, whose stability is already well known. Therefore, a minimum of six months stability was attributed both to standards and QCs.

## Preliminary experiments: recovery from the Salivette®

Preliminary experiments have been performed, in order to detect drug retention by Salivette^®^ matrix, using both saliva samples and solvent.

Three mix of solvent containing all sixteen drugs at 3 different concentrations (1000, 100 and 10 ng/mL) were prepared by independently spiking drug stock solutions in a mixture of water and acetonitrile (H2O:ACN 90:10, v:v) added with 0.05% of formic acid. Two mL of each solution were allowed to soak into the Salivette® cotton roll (performed in double replicate), followed by centrifugation and collection. A small amount (7 µL) of the resulting volume was directly injected in the UHPLC instrument and analysed together with the same volume deriving directly from the 3 mix, without passing through the Salivette^®^. In saliva samples, experiments have been focused on the 100 ng/mL concentration: blank sputum from healthy donors was collected in falcon tubes and then spiked with all the 16 drugs at the same concentration. Then, 2 mL were allowed to soak into the Salivette^®^ cotton roll (performed in double replicate), and then centrifuged, collected and extracted. The remaining volume was directly extracted without passing through the Salivette^®^.

Resulting peak areas have been compared to address the extent of the retention. In order to find possible correlations, a series of molecule-related parameters was collected for each drug: partition coefficient (logP), acid dissociation constant (pKa), molecular weight and chromatographic retention time.

# Supplementary tables

**Table 1s. Chromatographic gradient of mobile phases A (water + formic acid 0.05%) and B (acetonitrile + formic acid 0.05%).**

| **Time (min)** | **Flow (mL/min)** | **Mobile phase A (%)** | **Mobile phase B (%)** |
| --- | --- | --- | --- |
| 0.00 | 0.400 | 90 | 10 |
| 1.10 | 0.400 | 90 | 10 |
| 9.00 | 0.400 | 25 | 75 |
| 9.60 | 0.450 | 5 | 95 |
| 10.60 | 0.450 | 5 | 95 |
| 10.65 | 0.450 | 90 | 10 |
| 10.90 | 0.450 | 90 | 10 |
| 11.00 | 0.400 | 90 | 10 |
| 13.00 | 0.400 | 90 | 10 |

**Table 2s. Summary of instrument settings and multiple reaction monitoring transitions**.

| **GENERAL DETECTOR SETTINGS** | | | | | | | | | |
| --- | --- | --- | --- | --- | --- | --- | --- | --- | --- |
| **Drying Gas Temperature** | | | | | 130°C | | | | |
| **HSID Temperature** | | | | | 270°C | | | | |
| **Nebulizer Gas** | | | | | 350 | | | | |
| **ElectroSpray V1 Positive** | | | | | 5000 | | | | |
| **Source Temperature** | | | | | 350°C | | | | |
| **Multipole 1 RF** | | | | | 370 | | | | |
| **Collision Pressure** | | | | | 410 | | | | |
| **ANALYTE SPECIFIC PARAMETERS** | | | | | | | | | |
| **MASS PARAMETERS** | **DRUGS** | | | | | | | | |
|  | **CLN** | **DOX** | **AML** | **HCTZ** | | **NFD** | **TEL** | **RAM** | **OLM** |
| Primary Ion Trace (m/z) | 230.0>44 | 452.2>344.2 | 409.15>238.1 | 296>205.0 | | 347.15>239.1 | 515.25>211.1 | 417.2>117.1 | 447.2>207.1 |
| Collision Energy | -35 | -41 | -19 | 31 | | -20 | -55 | -53 | -30 |
| Entrance voltage | 41 | 47 | 10 | -40 | | 9 | 19 | 21 | 21 |
| Collision Cell Lens 2 | -60 | -116 | -76 | 88 | | -72 | -184 | -96 | -96 |
| Secondary Ion Trace (m/z) | 230.0>213 | 452.2>247.1 | 409.15>294.1 | 296>269.05 | | 347.15>195.1 | 515.25>276.2 | 417.2>234.2 | 447.2>235.1 |
| Collision Energy | -35 | -54 | -17 | 26 | | -49 | -62 | -27 | -29 |
| Entrance voltage | 39 | 50 | 9 | -40 | | 9 | 39 | 30 | 20 |
| Collision Cell Lens 2 | -92 | -160 | -84 | 72 | | -88 | -200 | -80 | -108 |
| Ionization | ESI+ | ESI+ | ESI+ | ESI- | | ESI+ | ESI+ | ESI+ | ESI+ |
|  | | | | | | | | | |
|  | **ATE** | **CHL** | **IDP** | **VAL** | | **NBV** | **SCB** | **SCB-M** | **RAM-M** |
| Primary Ion Trace (m/z) | 267.15>145.05 | 337.05>190 | 366.1>132.1 | 436.25>235.1 | | 406.2>123.1 | 412.2>193.1 | 384.2>193.15 | 389.2>117.1 |
| Collision Energy | -36 | 24 | -20 | -27 | | -57 | -44 | -40 | -50 |
| Entrance voltage | 11 | -32 | 18 | 18 | | 26 | 24 | 6 | 27 |
| Collision Cell Lens 2 | -64 | 76 | -68 | -80 | | -104 | -80 | -80 | -80 |
| Secondary Ion Trace (m/z) | 267.15>190.1 | 337.05>146 | 366.1>117.1 | 436.25>207.1 | | 406.2>151.1 | 412.2>266.2 | 384.2>266.2 | 389.2>206.15 |
| Collision Energy | -24 | 26 | -64 | -37 | | -39 | -25 | -24 | -28 |
| Entrance voltage | 8 | -35 | 11 | 18 | | 34 | 25 | 16 | 25 |
| Collision Cell Lens 2 | -64 | 64 | -76 | -84 | | -88 | -76 | -76 | -84 |
| Ionization | ESI+ | ESI- | ESI+ | ESI+ | | ESI+ | ESI+ | ESI+ | ESI+ |
| HSID, Heated Surface Induced Desolvation; RF RadioFrequency; CLN, Clonidine; DOX, Doxazosin; AML, Amlodipine; HCTZ, Hydrochlorothiazide; NFD, Nifedipine; TEL, Telmisartan; RAM, Ramipril; OLM, Olmesartan; ATE, Atenolol; CHL, Chlortalidone; IDP, Indapamide; VAL, Valsartan; NBV, Nebivolol; SCB, Sacubitril; SCB-M, Sacubitrilat; RAM-M, Ramiprilat; ESI, Electrospray ionization mode. | | | | | | | | | |
